# Determinación de *BRAF V600E* por biopsia líquida en oncología de precisión

**DOI:** 10.1515/almed-2024-0167

**Published:** 2025-07-03

**Authors:** Verónica A. Alonso, Gabriela R. Mendeluk

**Affiliations:** Departamento de Bioquímica Clínica, Hospital de Clínicas”José de San Martín”, Universidad de Buenos Aires, Instituto de Fisiopatología y Bioquímica Clínica- INFIBIOC, C.A.B.A, Argentina

**Keywords:** *BRAF V600E*, biopsia líquida, oncología de precisión, cáncer, tratamiento personalizado

## Abstract

**Introducción:**

La mutación *BRAF V600E* es una de las alteraciones genéticas más relevantes en oncología de precisión, comúnmente encontrada en varios tipos de cánceres como el melanoma, el cáncer colorrectal y cáncer de tiroides entre otros. La biopsia líquida permite detectar esta mutación de manera mínimamente invasiva.

**Contenido:**

Esta revisión analiza la biología molecular de la mutación *BRAF V600E*, su papel en la progresión tumoral, y su valor para el diagnóstico, pronóstico y tratamiento de cánceres específicos. Además, se exploran los métodos de detección mediante biopsia líquida y sus ventajas frente a la biopsia sólida.

**Resumen:**

La detección de *BRAF V600E* a través de biopsia líquida proporciona una alternativa menos invasiva que las biopsias tradicionales, valiosa para ajustar terapias y evaluar la respuesta al tratamiento.

**Perspectiva:**

Se espera que el uso de biopsia líquida para identificar *BRAF V600E* continúe difundiéndose en los distintos centros de salud, optimizando así el seguimiento y tratamiento de los pacientes oncológicos con enfoques personalizados.

## Introducción

La oncología de precisión consiste en la detección de alteraciones genéticas que juegan un rol central en la oncogénesis, para orientar tratamientos específicos y efectivos a cada paciente [1]. También es utilizada para el monitoreo y pronóstico de ciertos tipos de cánceres. Los avances en el análisis genómico, la búsqueda y hallazgo de nuevos biomarcadores así como el desarrollo de fármacos y el diseño innovador de ensayos clínicos, hacen que la oncología de precisión se torne imprescindible en el manejo del cáncer [1], [2].

Una de las mutaciones conductoras más importantes en muchos cánceres es *BRAF V600E* (mutación que ocurre en el exón 15 del gen *BRAF,* con un cambio de timina por adenina en el nucleótido 1799, que conduce a una sustitución de valina por ácido glutámico en el codón *600*) [3].

*BRAF* (homólogo B del oncogén viral del sarcoma murino v-raf) es una serina-treonina quinasa regulada por *RAS* que actúa como un activador en la cascada de señalización de la vía dependiente de la proteína quinasa activada por mitógenos *MAPK* (*RAS- RAF- MEK- ERK*). Esta vía puede ser activada por el receptor del factor de crecimiento epidérmico (*EGFR*) y en condiciones normales conduce a la proliferación celular, pero ante la presencia de una mutación de ganancia de función en oncoproteínas como *BRAF* favorece el desarrollo de neoplasias [3]. Cabe aclarar que *BRAF* puede tener distintos tipos de mutaciones clasificadas en clase I, II y III. *BRAF V600E* es la mutación de clase I más común en oncología, siendo *BRAF V600 K/D/R* también de la misma clase pero menos frecuentes [4]. Las mutaciones *BRAF no V600* se pueden clasificar en clase II y III [4].

La clase II tiene una mutación en el segmento activo del dominio quinasa (*K601 E/N/T, L597V/Q/R*), y anillo P (*G464 V/E, G469 A/V/R*) [4].

La clase III tiene mutado también el anillo P al igual que en la clase II pero la mutación es *G466 A/E/V*. También puede ocurrir en el anillo catalítico (N581I/S/T) y en el motivo DGF del dominio quinasa (*D594 A/G/H/N, D596D/R*) [4].

Existen otras mutaciones posibles de *BRAF* menos frecuentes no incluidas en la clasificación I, II y III. Se trata de deleciones en el segmento corto espiral α-C del dominio quinasa faltando todo el marco de lectura o de fusiones con pérdida de la porción N terminal [4].

Las mutaciones *BRAF* de clase I, específicamente *BRAF V600E*, tienen una alta frecuencia en tumores como el melanoma, cáncer colorrectal y cáncer de tiroides [4]. En pacientes con cáncer de pulmón y colorrectal con resistencia a los inhibidores del *EGFR*, se suelen detectar mutaciones BRAF de clase I y no dependen de la actividad de *RAS*, mostrando sensibilidad a los inhibidores de *BRAF* (*BRAFis*) y a los inhibidores de MEK o combinación de ambos [4].

Las mutaciones BRAF de clase II son independientes de la actividad de RAS y son sensibles a los *BRAFis*, inhibidores *pan-RAF*, inhibidores de *MEK* o a una combinación de los mismos [4]. En pacientes con cáncer de pulmón de células no pequeñas (CPCNP) con mutaciones del *EGFR*, las mutaciones *BRAF* de clase II a menudo se consideran parte fundamental para la resistencia adquirida a los inhibidores del *EGFR* [4].

Las mutaciones de *BRAF* de clase III dependen de la actividad de RAS y en ausencia de mutaciones *RAS* o *NF1* concurrentes, los tumores son sensibles a la inhibición de receptores tirosina kinasa y se asocian con una mayor sensibilidad a los inhibidores de *EGFR* y una supervivencia prolongada después del tratamiento en pacientes con cáncer colorrectal, diferenciándose así de las mutaciones *BRAF* de clase I, que son de mal pronóstico en este tipo de pacientes [4].

También pueden existir otras variantes menos frecuentes como deleciones o fusiones en el gen *BRAF,* las cuales no dependen de la actividad de RAS y tienen un mecanismo parecido al de las mutaciones de clase II [4].

Varios estudios sugieren que la inhibición de MEK es una estrategia activa en tumores con mutaciones *BRAF* fuera del locus V600 y en fusiones *BRAF*, por lo que se evaluó en un subprotocolo de NCI-MATCH del Instituto Nacional del Cáncer, la eficacia del inhibidor de *MEK* (trametinib) en pacientes con tumores sólidos y linfomas positivos para *BRAFV600E,* concluyendo que si bien no hubo actividad sustancial en esta población, se están desarrollando nuevos agentes dirigidos a la señalización de *MAPK*, como los inhibidores de ERK que ha mostrado una actividad clínica moderada en otros estudios [5].

Sintetizando los conceptos anteriores, podemos decir que conocer la clase de mutación de *BRAF* orienta al pronóstico y tratamiento dirigido.

Si bien se han descrito las otras alteraciones genéticas antes mencionadas, la literatura se ha focalizado en el estudio de *BRAF V600E*, identificándose como un biomarcador clave para el diagnóstico, pronóstico y seguimiento en diversos tipos de cánceres [3].

Nos proponemos hacer un relevamiento de la literatura para revelar las principales aplicaciones clínicas de la detección de *BRAF V600E* mediante biopsia líquida en oncología.

Hemos decidido seleccionar para la revisión de *BRAF V600E,* focalizarnos en el melanoma, el carcinoma papilar de tiroides y el cáncer colorrectal por su frecuencia de aparición, valor pronóstico y disponibilidad de terapias dirigidas, ya que han demostrado impacto clínico, pudiendo de esta manera personalizar el tratamiento, mejorar los resultados y avanzar en la oncología de precisión [4]. A continuación se detallarán los mismos y se mencionarán además otros tumores en los cuales dicha mutación es relevante.

## Melanoma

El 50 % de los melanomas metastásicos tienen mutaciones puntuales en BRAF, como *BRAF V600E* [6], [7]. Los *BRAFis* como vemurafenib y dabrafenib, son altamente efectivos en el melanoma avanzado positivo para *BRAF V600E*, con una tasa de respuesta de aproximadamente 60–80 % [8]. Se suelen utilizar en combinación con inhibidores de *MEK* (cobimetinib, trametinib, binimetinib) [6]. También se utilizan como parte de la terapia adyuvante luego de la resección quirúrgica para reducir el riesgo de recurrencia. Esto fue demostrado en un ensayo clínico de fase 3, realizado en pacientes a los que se les había resecado un melanoma en estadio III con mutaciones *BRAF V600E* o *V600K* con 12 meses de terapia adyuvante (dabrafenib + trametinib) en los que se logró una mayor sobrevida libre de enfermedad [9]. En un ensayo clínico en fase 2 prospectivo y multicéntrico se analizaron pacientes tratados con encorafenib (inhibidor de *BRAF*) y binimetinib (inhibidor de MEK) seguidos de radioterapia en pacientes con melanoma positivos para *BRAF V600* y con metástasis cerebrales. Se concluyó que con este tratamiento se logró un efecto clínico positivo en términos de tasa de respuesta intracraneal, con un perfil de seguridad aceptable y una baja incidencia de eventos adversos graves [10].

El ensayo clínico en fase 3 DREAMseq propone la combinación de nivolumab/ipilimumab seguido en caso de progresión, del tratamiento con inhibidores de *BRAF* y *MEK* (dabrafenib + trametinib) [11].

## Cáncer de tiroides

El carcinoma papilar de tiroides (CPT) es el tipo histológico más común de carcinoma diferenciado de tiroides (75–85 % de los casos) y se caracteriza por una baja tasa de mortalidad y una buena respuesta a la terapia con yodo radiactivo. Los distintos tipos de cánceres de tiroides suelen presentar diferentes alteraciones genéticas. El desarrollo de CPT está estrechamente relacionado con mutaciones puntuales somáticas en BRAF y reordenamientos en los genes RET/PTC1, RET/PTC3 y NTRK1/3. Curiosamente, las aberraciones de los genes impulsores en el cáncer de tiroides bien diferenciado son mutuamente excluyentes [12]. Los países occidentales han informado durante la última década la prevalencia de la mutación *BRAF V600E* en los EE. UU. y Europa, siendo entre 35 y 60 %. Las series asiáticas demostraron una considerable heterogeneidad entre y dentro de los países con respecto a la prevalencia de esta mutación en CPT [12].

Es sabido que la mutación del gen *BRAF V600E* se relaciona con una mayor resistencia al tratamiento con yodo radiactivo posquirúrgico [13]. En un ensayo multicéntrico, aleatorizado, abierto, de fase 2 se pudo concluir que la combinación de dabrafenib + trametinib no logró ser superior en eficacia a la monoterapia con dabrafenib en pacientes con cáncer de tiroides progresivo refractario al yodo radiactivo con la mutación en *BRAF* [14].

Cuando la frecuencia alélica de la mutación se encuentra aumentada en el carcinoma papilar de tiroides, el tumor resulta más agresivo, presenta mayor tamaño y número de metástasis en ganglios linfáticos [15]. La FDA aprobó la combinación de dabrafenib + trametinib para el cáncer anaplásico de tiroides así como también para el melanoma avanzado basándose en el ensayo clínico ROAR [16].

## Cáncer colorrectal

Se conoce que las mutaciones *BRAF V600E* se encuentran presentes en aproximadamente el 10 % de los casos de cáncer colorrectal metastásico siendo indicador de mal pronóstico [17]. Su hallazgo predice resistencia a los anticuerpos monoclonales anti-EGFR [17]. Es sabido que las mutaciones *BRAF V600E* en edad avanzada, tumor primario de colon derecho y sexo femenino, se asocian a mala respuesta a la quimioterapia y mal pronóstico [18]. Además, dichas mutaciones están estrechamente relacionadas con una baja expresión de las enzimas de reparación por apareamiento erróneo (MMR: mismatch repair) y con alta inestabilidad de microsatélites (*MSI-H*), por lo que resulta de vital importancia realizar un perfil molecular en todos los cánceres colorrectales metastásicos, ya que esta asociación tiene repercusiones tanto pronósticas como terapéuticas [18], [19].

En un estudio prospectivo de fase 3 realizado en pacientes con cáncer colorrectal metastásico que presentaron MSI-H, el tratamiento de primera línea con un anticuerpo monoclonal dirigido contra PD-1 (pembrolizumab) resultó en una supervivencia libre de progresión más larga con menos efectos adversos que los tratados con quimioterapia [20].

S CAPSTAN CRC, un estudio europeo observacional retrospectivo analizó tratamientos de cáncer colorrectal metastásico con mutación BRAF V600E tratados en un periodo de 4 años, observando que el tratamiento de elección fue quimioterapia (FOLFOX: 5-FU, oxaliplatino y leucovorina) más Anti-VEGF (bevacizumab). El inhibidor de BRAF más cetuximab se administró en muy pocos pacientes y en tercera línea [21]. Basándose en los resultados obtenidos en un ensayo de fase 3 prospectivo BEACON CRC, realizado en pacientes con cáncer colorrectal en estadío metastásico, la FDA ha aprobado la terapia doble con anti-EGFR y un inhibidor de BRAF (cetuximab + encorafenib) [22]. Según los resultados obtenidos en el ensayo clínico BREAKWATER, la combinación de la terapia dirigida dual (encorafenib y cetuximab) con quimioterapia en el *CCRm* con mutación *BRAF V600E* mejora los resultados de los pacientes como tratamiento de primera línea [22]. La combinación de quimioterapia citotóxica (que tiene un efecto antitumoral no selectivo) y terapia dirigida, puede superar la heterogeneidad intratumoral a través de un efecto aditivo, dirigiéndose a diferentes poblaciones celulares y, en última instancia, mejorando los resultados clínicos [22].

Aún con esta estrategia, la respuesta a los tratamientos sigue siendo baja en algunos pacientes. Por este motivo se han ensayado otras combinaciones de drogas, por ejemplo en un ensayo clínico de fase 2 se realizó una inhibición combinada de PD-1 (espartalizumab), BRAF (dabrafenib) y MEK (trametinib) fortaleciendo así la respuesta inmune que suele estar alterada en muchos tumores [23].

En la Tabla I se muestran las principales aplicaciones clínicas de *BRAF V600E* en la clínica oncológica en los tres tipos de tumores mencionados.

**Tabla I:** Principales aplicaciones clínicas de la detección de la mutación BRAF V600E en los tumores sólidos más relevantes.

Tipo de tumor sólido	Aplicaciones clínicas	
	Pronóstico	Terapia dirigida/Inmunoterapia
Melanoma [6], [7], [8, 11]	Mejora la tasa de respuesta al tratamiento dirigido en melanoma mestastásico o irresecable.	En caso de progresión, se recomiendan inhibidores de BRAF (ej: dabrafenib + trametinib) luego de la inmunoterapia (nivolumab + ipilimumab).
Cáncer colorrectal metastásico [20], [21], [22]	Se asocia a un pronóstico desfavorable.	Combinación de drogas inhibidoras de EGFR y BRAF (ej: cetuximab + encorafenib). En caso de MSI-H, se recomienda inmunoterapia (ej: pembrolizumab).
Cáncer de tiroides [13], [15], [16]	Se asocia a un mayor riesgo de recurrencia y extensión extratiroidea en el carcinoma papilar de tiroides con menor captación de yodo radioactivo.	Inhibidores de BRAF y MEK (dabrafenib + trametinib).

Se sintetizan los resultados de los principales estudios y ensayos clínicos de BRAF V600E.

## Otros tumores positivos para la mutación *BRAF V600E*

El cáncer de pulmón de células no pequeñas (*CPCNP*) es una de las principales causas de mortalidad a nivel mundial, en el que las mutaciones en *BRAF* son poco frecuentes (0 y 3 %). En los pocos casos positivos para la mutación en BRAF, se ha observado una mejor respuesta a la terapia combinada con inhibidores orales de *BRAF* y *MEK* [24]. *Dicha* mutación es indicadora de mal pronóstico y se recomienda la terapia combinada de dabrafenib y trametinib para los pacientes con estadío metastásico [25]. En el CPCNP también pueden ocurrir fusiones de BRAF, pero en estos casos existe una falta de estrategias terapéuticas efectivas. En este artículo, revisamos las características clínicas, el mecanismo de acción y el tratamiento clínico de la fusión de BRAF para sentar las bases para el tratamiento de la fusión de BRAF en pacientes con CPNM [25].

En referencia a los tumores del sistema nervioso central con dicha mutación, recientemente se ha reportado la utilidad de la terapia dirigida a los componentes de la vía de las MAPK [26]. Recientemente se ha demostrado que dabrafenib y su combinación con trametinib resultan eficaces en glioma pediátrico con mutaciones *BRAF* V600, lo que justifica evaluar esta combinación como terapia de primera línea [27].

En cuanto al cáncer de ovario seroso de bajo grado, los inhibidores de *BRAF* como agentes únicos fueron aprobados para el tratamiento de tumores con mutación *BRAF*. En algunos casos, la combinación de inhibidores de *BRAF* con inhibidores de *MEK* retrasa la aparición de resistencia en comparación con el inhibidor de *BRAF* como agente único [28].

En el ensayo clínico multicétrico ROAR fase 2, la combinación de dabrafenib con trametinib demostró actividad clínica tumoral-agnóstico en pacientes con cánceres mutados *BRAFV600E* raros, incluyendo carcinoma anaplásico de tiroides, cáncer de vías biliares, tumor del estroma gastrointestinal, adenocarcinoma de intestino delgado, glioma de bajo grado, glioma de alto grado, leucemia de células pilosas y mieloma múltiple [29].

## Utilización de biopsia líquida para la detección de BRAF V600E en ADN circulante tumoral

La biopsia líquida es una herramienta esencial que permite detectar diversos biomarcadores en la sangre o fluidos corporales de pacientes oncológicos mediante tecnologías de biología molecular altamente sensibles como la *qPCR* (PCR cuantitativa o en tiempo real), *dPCR* (PCR digital) o *NGS* (secuenciación de nueva generación), proporcionando una representación precisa del tejido de origen tumoral [30].

Con respecto al *cfDNA* (ADN libre de la fracción celular), éste es uno de los analitos extraídos más investigados en el contexto de la biopsia líquida. En general, los niveles de *cfDNA* son más altos en pacientes con cáncer que en individuos sanos, y tienden a incrementarse con el avance de la enfermedad y la presencia de metástasis, debido a la gran liberación de material genético de las células tumorales [30]. Sin embargo, los niveles elevados de *cfDNA* no son exclusivos de los tumores, ya que pueden aumentar en situaciones patológicas como no patológicas, tales como el ejercicio extenuante o luego de una cirugía. Esto dificulta la asociación directa con el cáncer, ya que el *cfDNA* también se encuentra en personas saludables y puede provenir de células no tumorales. De hecho, la mayor parte del *cfDNA* en circulación tiene su origen en células no malignas, siendo muchas de ellas de origen germinal [30], [31].

El *ctDNA*, que forma parte del *cfDNA*, es específico del tumor ya que se origina en células malignas por procesos de necrosis o apoptosis, y contiene las mutaciones somáticas características del tumor, reflejando así su dinámica [32], [33]. Dado que no se encuentra en personas sanas, el *ctDNA* permite identificar mutaciones accionables y evaluar la evolución del cáncer. Tanto el cfDNA como el *ctDNA* y las células tumorales circulantes (*CTC*) se asocian a la progresión de la enfermedad. Si bien los dos primeros presentan bajas concentraciones en sangre periférica resulta más probable hallarlos que a las *CTCs* [33]. Por este motivo, la mutación *BRAF V600E* se puede detectar en el *ctDNA*, tomando la precaución de almacenar las muestras de plasma a −80° si se guardara por largo plazo o a −20° si se procesara inmediatamente el *ADN* extraído [32], [33].

Una vez extraído el *ADN* se procede a su cuantificación. Si bien la espectrofotometría suele utilizarse para determinar de manera rutinaria las concentraciones de los ácidos nucleicos, así como su pureza, posee una desventaja y es que no es selectiva para *ADN*, ya que también detecta otros compuestos como *ARN* y proteínas, sobreestimando así la concentración de *ADN* [34]. Por este motivo es que el método de elección es el fluorimétrico, el cual utiliza fluoróforos intercalantes unidos específicamente al ácido nucleico a analizar [34].

## Técnicas de detección utilizadas

La cantidad total de *ctDNA* es sólo una mínima parte del *cfDNA.* Concentraciones tan bajas sólo pueden ser medidas por métodos muy sensibles, particularmente para estadíos tempranos del tumor [35]. Las técnicas mayormente utilizadas son PCR cuantitativa o en tiempo real (*qPCR*), *PCR digital* (*dPCR*) y Secuenciación de nueva generación (*NGS*) [35].

La *dPCR* se caracteriza por su elevada sensibilidad. Divide a la muestra en miles de reacciones individuales en pocillos donde cada uno puede tener 0, 1 o más copias del ADN de interés [36]. Así se pueden identificar variantes poco frecuentes pero conocidas y detectar cambios sutiles en el número de copias. Esta técnica es capaz de detectar hasta 1 copia mutante entre 10,000, equivalente a un *MAF* (frecuencia del alelo menor) de 0.01 %. En contraste, métodos como la *qPCR* analizan todas las señales al mismo tiempo, lo que puede ocultar aquellas de menor intensidad, con una *MAF* de hasta 0.1 % [36], [37].

Otra característica clave de la *dPCR* es su capacidad para realizar una cuantificación absoluta [36]. A diferencia de la *qPCR* que puede realizar una cuantificación relativa, la dPCR reduce la probabilidad de falsos positivos y, en el contexto de la biopsia líquida, minimiza el riesgo de falsos negativos, especialmente cuando la cantidad de ADN disponible es limitada y las mutaciones se encuentran en baja frecuencia [36].

*NGS* es capaz de detectar *MAF*<1 % y posee la ventaja de que puede analizar variantes desconocidas a diferencia de *qPCR* o *dPCR* en las cuales se debe conocer la variante a analizar [37].

## Ventajas de la biopsia líquida sobre la biopsia sólida para la determinación de BRAF V600E

– **Monitorización no invasiva de la enfermedad:** La biopsia líquida permite el seguimiento continuo de mutaciones como *BRAF V600E* a través de muestras de sangre, evitando la necesidad de biopsias sólidas repetitivas. Esto es especialmente útil en tumores de difícil acceso o en pacientes con enfermedad avanzada, donde el análisis de *ctDNA* puede reflejar cambios en tiempo real y orientar ajustes terapéuticos de manera más ágil [38].
– **Enfermedad mínima residual (EMR):** La detección de *BRAF V600E* mediante biopsia líquida facilita la identificación temprana de células tumorales persistentes tras el tratamiento [39]. A diferencia de la biopsia sólida, que solo analiza un fragmento estático del tumor, la biopsia líquida puede detectar enfermedad residual en todo el organismo, permitiendo una vigilancia más efectiva y otorgando la posibilidad de intervenciones terapéuticas precoces [39].
– **Heterogeneidad tumoral:** La biopsia líquida supera las limitaciones de la biopsia sólida al capturar la diversidad genética del tumor en su conjunto [40]. En el caso de *BRAF V600E*, esta técnica permite identificar la coexistencia de subpoblaciones tumorales con y sin la mutación, lo que resulta crucial para evaluar la resistencia a tratamientos dirigidos y diseñar estrategias terapéuticas más precisas y dinámicas [40].

## Validación y estandarización de biopsia líquida

Para incorporar la biopsia líquida en la práctica clínica, es esencial optimizar y estandarizar los protocolos de aislamiento de los analitos. Con el fin de acelerar el desarrollo, validación y aplicación clínica de esta técnica, existen organizaciones públicas y privadas en Europa y Estados Unidos (como *BloodPAC*), reuniendo la experiencia de la industria y la academia para establecer ensayos robustos y reproducibles [30].

## Ensayos comerciales para la detección de BRAF V600E mediante biopsia líquida

Existen varios kits comerciales para la detección de la mutación *BRAF V600E,* dirigidos al análisis de *ctDNA*. Algunos ejemplos son:

– Guardant 360 (Guardant Health): Es un panel de *ctDNA* que permite el análisis en distintos tipos de tumores como cáncer de pulmón, colorrectal y melanoma, utilizando NGS [41].
– FoundationOne Liquid (Foundation Medicine): Es un panel de biopsia líquida que detecta más de 300 genes relacionados con el cáncer, utilizando *NGS.* Es útil para el tratamiento y detección de recidivas [41].
– Therascreen *BRAF V600E* Kit (QIAGEN): Basado en *qPCR* [42].
– EntroGen *BRAF* Mutation Analysis Kit: basado en *qPCR* diseñado para detectar *BRAF V600E* [43].
– Idylla™ *BRAF* Mutation Test (Biocartis): Está basado en *qPCR* completamente automatizado [44].

## Discusión

La mutación *BRAF V600E* es una alteración genética que juega un rol clave en la progresión de varios tipos de cánceres. Su relevancia ha sido ampliamente documentada en oncología de precisión.

Si bien el foco central de esta revisión es el análisis de la mutación *BRAF V600E* detectada en el *ctDNA* en el contexto de biopsia líquida, resulta indispensable integrar evidencia proveniente de estudios realizados en tejido tumoral. Ésto se debe a que gran parte del conocimiento actual sobre el valor pronóstico de esta alteración así como la eficacia de los tratamientos dirigidos, proviene de ensayos clínicos que utilizaron muestras de tejido como estándar diagnóstico. La inclusión de esta evidencia nos parece fundamental para contextualizar el impacto clínico de la detección de esta mutación, adoptando un enfoque integrador que considera la información disponible en tejido como referencia clave para comprender el potencial y los desafíos de la aplicación de este biomarcador en biopsia líquida, particularmente cuando el acceso al tejido es limitado o se requiere un monitoreo dinámico de la enfermedad.

En este trabajo nos focalizamos prioritariamente en la importancia de su detección en el melanoma, el cáncer de tiroides y de colon ya que es una mutación conductora clave en dichos tipos de tumores, haciendo también mención a su utilidad en el CPCNP y otros tipos de tumores portadores de dicha mutación.

De todos los cánceres estudiados, es en el melanoma donde su utilidad ha demostrado ser realmente relevante. La alta frecuencia de esta mutación en dicho tumor la transforma en un blanco molecular de elección para la terapia dirigida. Los pacientes con estadío avanzado positivos para *BRAF V600E*, tienen mejor pronóstico ya que mejora la tasa de respuesta con el tratamiento personalizado.

A diferencia del melanoma, tanto en el cáncer colorrectal como en el de tiroides, el hallazgo de la mutación en estudio tiene mal pronóstico y es entonces cuando los ensayos clínicos prueban terapias combinadas. Así en el caso de cáncer de colon se suman inhibidores de *EGFR, BRAF*, quimioterapia e inmunoterapia. Tratándose del cáncer de tiroides se ensayan Inhibidores de *BRAF* y *MEK*.

En cuanto a la técnica de detección en biopsia líquida para detectar *BRAF V600E* en ctDNA, concluimos que la elección de la misma dependerá de la sensibilidad requerida y de si se conoce o no la variante mutada. La *dPCR* estaría indicada en casos de tumores en estadíos tempranos, de sospecharse EMR o variantes alélicas poco frecuentes. En caso de querer hallar variantes desconocidas, la *NGS* sería la indicada. Aunque tiene costos elevados, con el tiempo estos disminuirán, lo que facilitará la recolección de una mayor cantidad de datos y contribuirá a un manejo más eficaz del cáncer [37].

La detección de *BRAF V600E* a través de biopsia líquida permite el seguimiento mínimamente invasivo de la enfermedad y la evaluación de la respuesta a terapias dirigidas, consolidando su importancia en medicina de precisión. En la práctica clínica es creciente el uso de biopsia líquida, la cual requiere de la utilización de tecnologías sensibles; sin embargo, aún existen aspectos importantes como las indicaciones adecuadas para su uso, limitaciones de la técnica como la sensibilidad, reporte de variantes, interpretación de resultados, relación costo-beneficio y cobertura en el Plan de Salud [30].

La biopsia líquida tiene diversas aplicaciones en la práctica oncológica, siendo el ctDNA el biomarcador más utilizado [30]. Esta técnica ha demostrado ser beneficiosa en diversas etapas del desarrollo tumoral ya que puede servir como herramienta diagnóstica tanto en las etapas tempranas como en las más avanzadas cuando ocurre la metástasis [30]. También es utilizada para complementar las biopsias de tejido y otros métodos diagnósticos y de seguimiento actuales. Además, ha facilitado la estadificación y seguimiento de la enfermedad, pudiendo detectar la enfermedad mínima residual (EMR) después de intervenciones quirúrgicas en diversos tipos de cáncer, y el monitoreo de la respuesta terapéutica [30], [45]. Es decir, permite evaluar la evolución temporal de un paciente que se somete a una cirugía o tratamiento inicial, predecir la recaída de la enfermedad y servir para guiar el tratamiento. Al principio, puede haber un foco de la enfermedad con una biologìa molecular característica, pero con el tiempo y la aparición de las metástasis, pueden surgir distintos clones, reflejando a diferencia de la biopsia de tejido o biopsia sólida, la heterogeneidad tumoral [45], [46].

### Conclusiones y perspectivas futuras

La evidencia reportada luego de una revisión exhaustiva de la bibliografía, indica que *BRAF V600E* es un biomarcador pronóstico y de seguimiento clave además de ser una importante diana terapéutica.

La detección de dicha mutación en melanoma es auspiciosa ya que existen terapias dirigidas como los inhibidores de BRAF y MEK que han mejorado notablemente la supervivencia de los pacientes que la portan.

En el cáncer colorrectal, su presencia se asocia a un pronóstico desfavorable con resistencia a terapias convencionales que redundan en una menor supervivencia global. Algo similar ocurre en el carcinoma papilar de tiroides donde el hallazgo de la mutación predispone a que el tumor sea más agresivo y presente mayor riesgo de recurrencia. Sin embargo, en ambos casos su detección es útil para definir estrategias terapéuticas personalizadas.

En síntesis, la identificación de esta mutación permite no solo estimar la evolución clínica de la enfermedad, sino también definir estrategias de tratamiento en el marco de la oncología de precisión.

Se trata de un método diagnóstico del que tenemos algunos resultados a partir de ensayos clínicos que aún no ha sido completamente transferido a la práctica oncológica. Entendemos que su detección mediante esta técnica arroja ventajas sobre la biopsia sólida, tales como la monitorización no invasiva de la enfermedad, la detección de enfermedad mínima residual y la representación de la heterogeneidad tumoral.

Las perspectivas futuras serían la estandarización de los protocolos de trabajo y el desarrollo de nuevas formulaciones dirigidas a componentes de la vía de señalización en estudio que podrían revelar alternativas aún desconocidas para mejorar la sobrevida y calidad de vida de los pacientes. La implementación de la detección de *BRAF V600E* en las instituciones de salud podría consolidarse a futuro como una herramienta clave en la oncología de precisión, optimizando así el diagnóstico y tratamiento de diversas neoplasias.

Nuestro objetivo fue conocer y difundir el valor clínico de la detección de *BRAF V600E* por biopsia líquida en oncología.
